# The ^1^H, ^15^N and ^13^C backbone resonance assignments of an intrinsically disordered region (467–696) of breast cancer type 1 susceptibility protein (BRCA1)

**DOI:** 10.1007/s12104-026-10277-2

**Published:** 2026-09-04

**Authors:** Hoang H. Dinh, Angela M. Jasper, Antoine Baudin, Patrick Sung, David S. Libich

**Affiliations:** 1 Department of Biochemistry and Structural Biology, and Greehey Children’s Cancer Research Institute, The University of Texas at San Antonio Health Science Center, 8403 Floyd Curl Dr., San Antonio, TX 78229 USA; 2 Present Address: Present address: Laboratoire de Chimie et Biochimie Pharmacologiques et Toxicologiques, Université Paris Cité, Paris, France; 3 Present Address: Present address: Laboratoire de Chimie de Coordination, CNRS, Toulouse, France

**Keywords:** BRCA1, BARD1, DNA repair, Homologous recombination, Intrinsically disordered protein, NMR

## Abstract

The tumor suppressor protein breast cancer type 1 susceptibility protein (BRCA1) plays a central role in maintaining genome stability through its involvement in DNA damage repair, transcriptional regulation, and cell-cycle control. BRCA1 functions as an obligate heterodimer with its binding partner, the BRCA1-associated RING domain protein 1 (BARD1), to coordinate accurate DNA repair. While the structured N- and C-terminal domains of BRCA1 have been well-characterized, the large central region encoded largely by exon 11 that comprises ~ 80% of the protein, is intrinsically disordered, and remains poorly structurally characterized. This intrinsically disordered region (IDR) harbors critical interaction interfaces for key proteins involved in genome maintenance, including RAD50, RAD51, MYC, and RB. Here, we report the backbone resonance assignments of a BRCA1 IDR construct spanning residues 467–696, providing a foundation for future studies aimed at understanding how the disordered central region of BRCA1 contributes to homologous recombination, interactions with BARD1, and overall BRCA1 tumor suppressor function.

## Biological context

The tumor suppressor breast cancer type 1 susceptibility protein (BRCA1) plays a critical role in maintaining genome stability (Miki et al. 1994). Germline mutations in *BRCA1* are strongly associated with an increased risk of breast, ovarian, pancreatic, and prostate cancers (Friedman et al. 1994; Pilarski 2019). Functionally, the BRCA1 protein participates in diverse cellular processes, including transcriptional regulation, cell-cycle checkpoint control, and DNA damage repair (Stefansson and Esteller 2012). These functions are largely mediated by the ability of BRCA1 to interact with numerous biomolecular partners through its multiple functional domains (Tarsounas and Sung 2020). At its N-terminus, BRCA1 forms an obligate heterodimer with BARD1 (BRCA1-associated RING domain protein 1) via their RING domains, conferring E3 ubiquitin ligase activity to the complex. This activity has been reported to be important for DNA repair, while it appears dispensable for replication fork maintenance (Densham et al. 2016). Further studies have shown that the BRCA1-BARD1 E3 ligase is important for DNA end resection, the initiating step of homologous recombination (HR), as well as for later stages required to complete the repair process (Wang et al. 2023). Toward the C-terminus, a coiled-coil domain (residues 1397–1424) mediates BRCA1 association with BRCA2 through the adaptor protein PALB2, itself a tumor suppressor (Zhang et al. 2009). This interaction promotes the replacement of RPA with RAD51 on single-stranded DNA, a critical step in HR (Tarsounas and Sung 2020). The extreme C-terminus harbors two highly conserved BRCA1 C-terminal (BRCT) repeats (residues 1642–1736 and 1756–1855) that recognize phosphorylated proteins through the pSXXF motif, a feature essential for BRCA1 tumor suppressor function (Shakya et al. 2011; Ismail et al. 2024). These repeats mediate interactions with signaling and transcriptional regulators, including AKT kinase, RNA polymerase II, and transcription factors such as TP53 (p53) and CtIP, highlighting a role in transcriptional regulation and cell-cycle control (Ismail et al. 2024). The BRCT repeats of BRCA1 also recruit several DNA repair proteins, including ABRAXAS, BRIP1 (also known as FANCJ, BACH1), and CtIP, forming the BRCA1-A, -B, and -C complexes, respectively (Wang 2012), which mediate recruitment of BRCA1 to sites of DNA damage and promote the early steps of HR-mediated DNA repair (Cruz-Garcia et al. 2014; Zou et al. 2014; Wu et al. 2016).

Despite the critical roles of these terminal domains in BRCA1 function, they comprise less than 15% of the 1,863-residue protein. The majority of BRCA1, particularly the central region predicted to be largely intrinsically disordered, remains functionally uncharacterized. A substantial portion of this central region is encoded by exon 11, which accounts for approximately 60% of the protein. Notably, ~ 30% of pathogenic germline variants in BRCA1 occur within exon 11 (Thompson et al. 2002). Truncating variants in this region result in HR deficiency and sensitivity to PARP inhibitors, whereas overexpression of exon 11 splice variants can promote PARP inhibitor resistance (Wang et al. 2016; Nacson et al. 2020). Mechanistically, this intrinsically disordered region (IDR) of BRCA1 contains interaction interfaces for several key binding partners involved in genome maintenance, including RAD50 (Zhong et al. 1999), RAD51 (Scully et al. 1997), MYC (Zeller et al. 2006), and RB (Aprelikova et al. 1999). The BRCA1 IDR has also been reported to bind DNA (Paull et al. 2001), suggesting a potential direct role in BRCA1 function. However, the disordered nature of this region has limited structural characterization, thereby hindering a detailed understanding of its functional contributions. To address this gap, we initiated structural studies of the BRCA1 IDR in parallel with the BARD1 IDR to better define its role in BRCA1 function, particularly in HR. Here, we report the backbone resonance assignments of a BRCA1 IDR construct spanning residues 467–696 (BRCA1^467–696^), providing a foundation for future studies aimed at understanding how the disordered central region of BRCA1 contributes to homologous recombination and overall BRCA1 tumor suppressor function.

## Methods and experiments

### Expression and purification of BRCA1^467–696^

The plasmid encoding His_6_-SUMO-BRCA1^467–696^ in the pE-SUMO vector was transformed into *E. coli* BL21 Star (DE3) by heat shock at 42 °C. A single colony was used to inoculate 5 mL of lysogeny broth (LB) supplemented with 100 µg/mL of ampicillin and grown for 6–8 h at 37 °C. One milliliter of the starter culture was used to inoculate 100 mL of M9 medium supplemented with 100 µg/mL of ampicillin and either 1 g/L of ^15^NH_4_Cl, or 1 g/L of ^15^NH_4_Cl and 3 g/L of ^13^C_6_-glucose (Sigma Millipore) for ^15^N or ^15^N,^13^C labeling, respectively, and grown overnight at 37 °C. The following day, the overnight culture was added to 900 mL of M9 medium supplemented with 100 µg/mL of ampicillin and the appropriate isotopic sources, and incubated at 37 °C with shaking until the OD_600_ reached a value of 0.6–0.8. Protein expression was induced by the addition of 0.5 mM isopropyl β-D-1-thiogalactopyranoside (IPTG) to the culture medium and incubated for an additional 4 h. The bacteria were harvested by centrifugation at 4,000 ×g for 30 min at 4 °C, and cell pellets were frozen at -80 °C.

All purification steps were carried out at 0–4 °C. Bacterial pellets were thawed on ice and resuspended in buffer A (25 mM Tris-HCl pH 7.5, 10% glycerol, 1 mM TCEP, 0.5 mM EDTA, 0.05% IGEPAL CA-630) supplemented with Turbonuclease (Sigma, 500 units), protease inhibitors (5 µg/mL of aprotinin, chymostatin, leupeptin, and pepstatin), 2 mM MgCl_2_, 20 mM imidazole, and 500 mM KCl. Cells were lysed by sonication, and the lysate was clarified by centrifugation at 45,000 ×g for 45 min. The clarified lysate was incubated with 2 mL of Ni-NTA resin (Qiagen) for 1 h. The resin was washed with 100 mL of buffer A supplemented with 2 mM MgCl_2_, 1 mM ATP, 20 mM imidazole, and 1 M KCl, followed by 20 mL of buffer A containing 20 mM imidazole and 500 mM KCl. Bound protein was eluted from the Ni-NTA resin in five fractions of 2 mL each using buffer A supplemented with 200 mM imidazole and 500 mM KCl. Eluted fractions were pooled and incubated overnight at 4 °C with Ulp1 protease (1:500 Ulp1:BRCA1 w/w ratio) to cleave the His_6_-SUMO tag. The sample was buffer-exchanged into buffer A containing 500 mM KCl using an Amicon^®^ Ultra-15 centrifugal filter (Merck) with a 10 kDa molecular weight cutoff, then incubated with 0.5 mL of Ni-NTA resin for 30 min to remove the cleaved tag and protease. The flow-through containing tag-free BRCA1^467–696^ was collected, buffer-exchanged into NMR buffer (20 mM KH_2_PO_4_ pH 6.5, 150 mM KCl, 0.5 mM TCEP, 0.2 mM EDTA), and concentrated to 0.5 mL. The sample was loaded on a Superdex Increase 75 10/300 size exclusion chromatography column (Cytiva) equilibrated with NMR buffer as a final polishing step. Pure BRCA1^467–696^ was collected, concentrated to approximately 600 µL, aliquoted, flash frozen in liquid N_2_, and stored at -80 °C.

### NMR spectroscopy

^15^N,^13^C-BRCA1^467–696^ was reconstituted at 200 µM in 20 mM KH_2_PO_4_ pH 6.5, 150 mM KCl, 0.5 mM TCEP, 0.2 mM EDTA, with 1 mM phenylmethylsulfonyl fluoride (PMSF), 10% D_2_O, and 0.5 mM sodium trimethylsilylpropanesulfonate (DSS) for chemical shift referencing. All experiments were acquired at 298 K on a Bruker NEO spectrometer operating at a ^1^H Larmor frequency of 700.13 MHz, equipped with a Bruker 5 mm TCI z-axis gradient cryogenic probe.

^1^H and ^15^N chemical shifts were obtained from a ^1^H,^15^N HSQC spectrum acquired with 128* × 1024* complex data points in the indirect (^15^N) and direct (^1^H) dimensions, corresponding to acquisition times of 82.0 and 112.6 ms, respectively. Chemical shifts of the backbone carbon atoms (^13^Cα, ^13^Cβ and ^13^C’) were obtained from three-dimensional triple resonance experiments (Grzesiek and Bax 1992; Yamazaki et al. 1994) HNCO, HN(CA)CO, CBCA(CO)NH, and HNCACB (Bruker Topspin 4.1 pulse programs) recorded in non-uniform sampling (NUS) mode(Delaglio et al. 2017) with a sampling density of 25% for HNCO, HN(CA)CO, and CBCA(CO)NH experiments, and 20% for the HNCACB experiment. Spectra were acquired with the following parameters: HNCO and HN(CA)CO, 32* × 64* × 1024* complex points in the indirect (F1, ^13^C), (F2, ^15^N) and direct (F3, ^1^H) dimensions corresponding to acquisition times of 16.5, 41.0, 112.6 ms, respectively; CBCA(CO)NH and HNCACB, 128* × 64* × 1024* complex points in the indirect (F1, ^13^C), (F2, ^15^N) and direct (F3, ^1^H) dimensions corresponding to acquisition times of 12.5, 41.0, 112.6 ms, respectively. Water suppression was achieved through a WATERGATE pulse sequence scheme (Piotto et al. 1992).

Spectra were reconstructed from NUS data using the SMILE (Ying et al. 2017) algorithm implemented in NMRPipe, and subsequently all spectra were apodised with a shifted sine bell function and zero filled to twice the number of acquisition points. Spectra were referenced in ^1^H, ^13^C and ^15^N dimensions with respect to the DSS ^1^H methyl frequency (Wishart et al. 1995). All spectra were processed with NMRPipe (Delaglio et al. 1995) and resonance assignments were achieved using the CCPNMR Analysis 2.5 software package (Skinner et al. 2016).

## Extent of assignment and data deposition

**Fig. 1:**
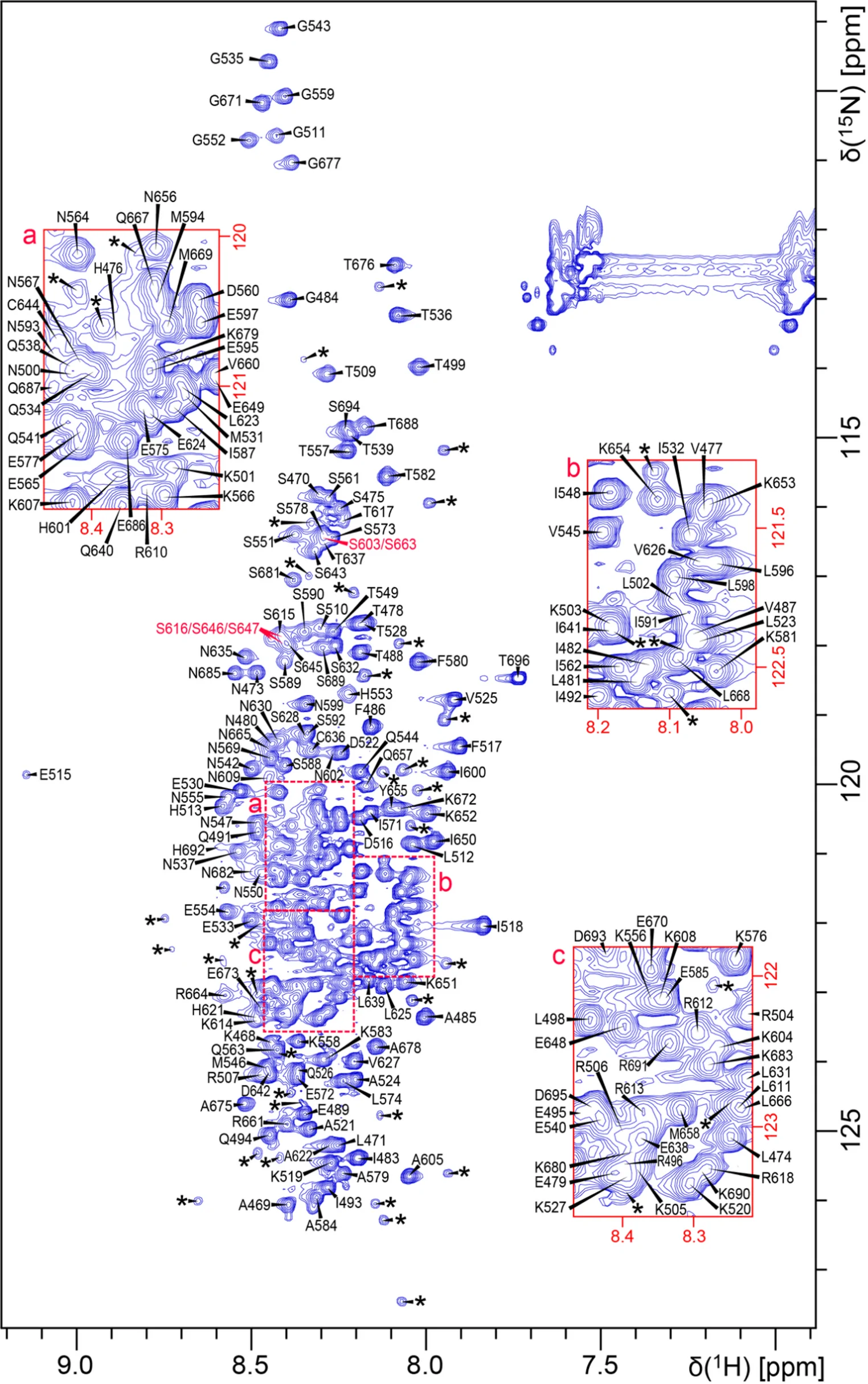
Assigned ^1^H,^15^N HSQC spectrum of BRCA1^467–696^. The spectrum was acquired at 298 K on a 200 µM sample of ^15^N,^13^C BRCA1^467–696^ dissolved in 20 mM KH_2_PO_4_ pH 6.5, 150 mM KCl, 0.5 mM TCEP, 0.2 mM EDTA, 1 mM PMSF, 10% D_2_O and 0.5 mM DSS. Resonances arising from asparagine and glutamine side chains are not labeled, ambiguously assigned peaks are labeled in red, and peaks that could not be unambiguously identified and/or assigned are denoted with an asterisk (*). Red dashed lines delineated the inset boundaries and are labeled with a corresponding **a**, **b**, and **c**

In the ^1^H,^15^N-HSQC spectrum of BRCA1^467–696^, 251 correlation peaks were observed, exceeding the 214 expected peaks (230 residues minus 15 prolines and the N-terminal residue, Fig. 1). Analysis of the 3D spectra yielded assignment of 205 residues, corresponding to 96% of the expected cross peaks in the ^1^H,^15^N-HSQC spectrum. Spin systems corresponding to residues H619, I620, R629, and H662 could not be identified. Peaks tentatively classified as serine residues but lacking unambiguous assignment due to sequence degeneracy, severe spectral overlap, and/or signal broadening in the ^13^C dimension are labeled in red (Fig. 1). Three such spin systems likely correspond to S616, S646, and S647, each occurring within a serine–serine pair whose chemical shift ambiguity precluded definitive assignment. Similarly, S603 and S663, preceded by arginine and lysine respectively, could not be unambiguously assigned and are also marked in red.

The remaining 43 unassigned peaks (denoted with *, Fig. 1) are more puzzling. Some may correspond to the four unobserved residues or arise from proline cis/trans isomerization (Alderson et al. 2018). Direct assignment was unsuccessful, as their ^13^C cross peak intensities were too low to reliably assemble spin systems; likewise, ^15^N *R*_2_ relaxation rates and ^3^*J*_HNHA_ coupling measurements yielded peak intensities too weak for reliable quantification. We purified the protein multiple times without detecting a co-purifying contaminant at a concentration sufficient to be visible by NMR, which we estimate would require at least 5–10% of the BRCA1^467–696^ concentration. ZZ-exchange experiments likewise revealed no cross peaks indicative of a second BRCA1^467–696^ conformation in slow exchange with the major state, although such slow exchange is generally less likely for an intrinsically disordered protein. We therefore speculate that these peaks arise either from a co-purifying contaminant present below our biochemical detection threshold or from an alternate conformation of BRCA1^467–696^ stabilized by an unidentified cofactor. Sample degradation could also account for a subset of these peaks, though no degradation products were observed on SDS-PAGE gels. Regardless of their origin, we are confident in the assignments reported here, which have enabled identification of binding sites for two BRCA1 biomolecular partners that were subsequently validated by mutational analysis in both in vitro and in vivo functional assays (Jasper et al. 2026). Further experimental characterization of BRCA1^467–696^ is ongoing. Overall, 96% of ^13^Cα, 96% of ^13^Cβ, 96% of ^13^C’, 92% of ^15^N, and 92% of ^1^H_N_ backbone resonances were assigned.

**Fig. 2:**
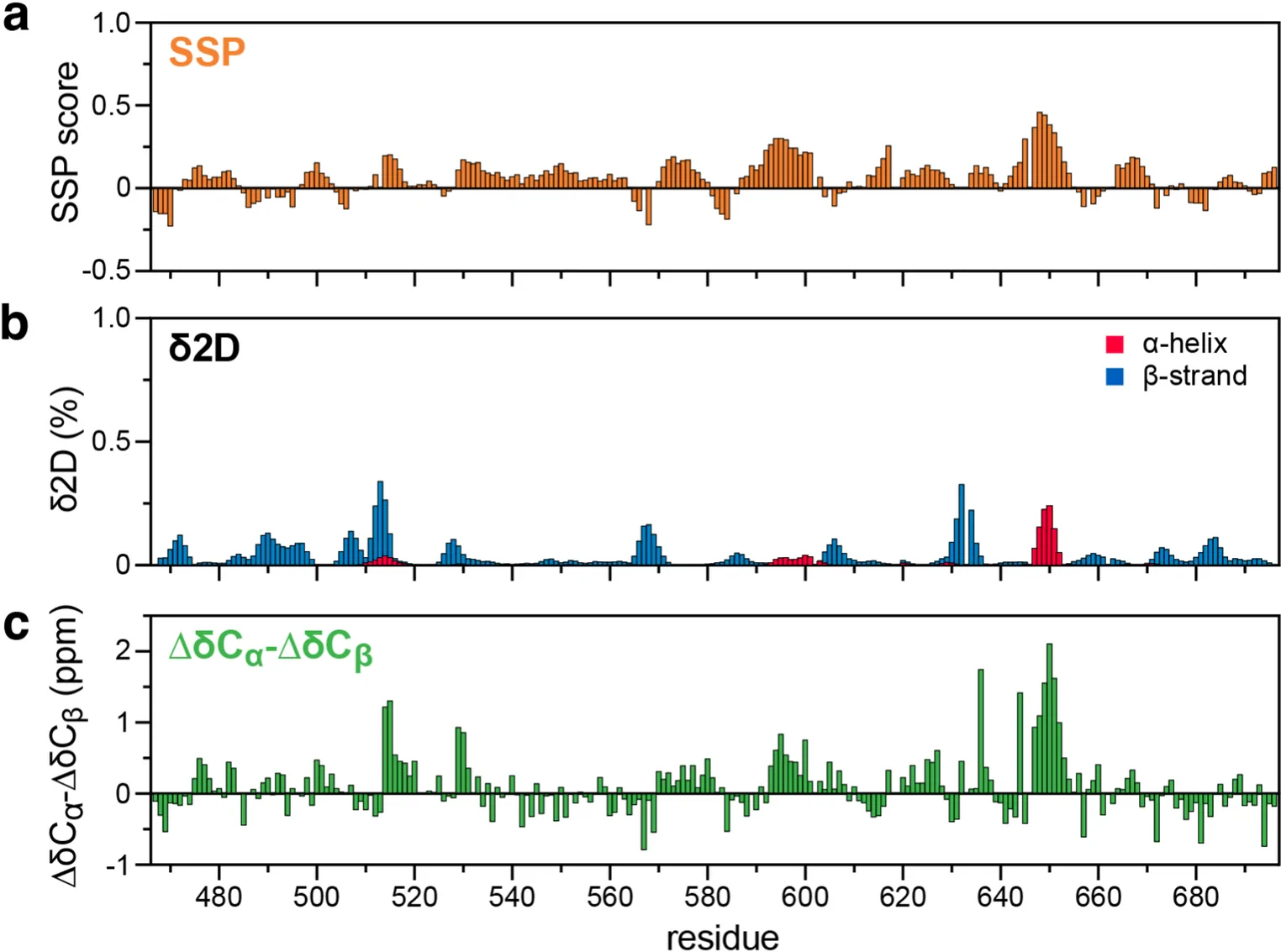
Secondary structure propensity of BRCA1^467–696^. (**a**) Secondary structure propensity (SSP) values calculated from backbone chemical shifts, where values closer to + 1 or -1 predict the propensity of α-helical or β-strand conformations, respectively. (**b**) δ2D score plotted against the protein sequence. Red and blue bars represent the probability score for the formation of α-helices and β-strands, respectively. (**c**) Δδ^13^Cα-Δδ^13^Cβ secondary chemical shifts (ppm) plotted as a function of the protein sequence. Positive values are representative of α-helices, negative values are representative of β-strands

Using the chemical shifts of the backbone and Cβ atoms, we calculated secondary structure propensities for BRCA1^467–696^ using the secondary structure propensity (SSP) (Marsh et al. 2006) and δ2D (Camilloni et al. 2012) algorithms, along with secondary chemical shift analysis (Δδ^13^Cα-Δδ^13^Cβ) (Kjaergaard and Poulsen 2011) (Fig. 2). An identical set of ^13^Cα, ^13^Cβ, ^13^C’, ^1^HN and ^15^N shifts from BRCA1 was used as input and both programs were run using their default settings, including the SSP weighted averaging window of 5 residues. SSP analysis revealed predominantly low scores across most of the sequence, with several regions showing modest positive values (generally below 0.25) indicative of weak helical propensity (Fig. 2a). These results suggest that the fragment is largely disordered while transiently sampling secondary structural elements. Notably, the region spanning residues 647–654 exhibited SSP scores of approximately 0.5, indicating a significantly higher helical propensity and suggesting that roughly half of the protomers comprising the conformational ensemble in this region adopts α-helical conformation(s). The δ2D analysis supports the structural tendencies identified by SSP, with the same region (residues 647–654) predicted to adopt α-helical conformation (Fig. 2b). To further evaluate the secondary structure propensity of this BRCA1 fragment, secondary chemical shifts (Δδ^13^Cα-Δδ^13^Cβ) were calculated using the experimentally determined ^13^Cα and ^13^Cβ chemical shifts and the corresponding random coil chemical shift values predicted from the protein sequence using the Poulsen IDP/IUP random coil chemical shifts web server (Kjaergaard and Poulsen 2011). Consistent with the SSP and δ2D analyses, most residues within BRCA1^467–696^ display secondary chemical shift values close to 0 ppm, indicating a lack of stable secondary structure (Fig. 2c). Several regions exhibit modest positive Δδ values (~ 1 ppm), suggesting transient helical propensity. Notably, residues 647–654 show Δδ values of up to ~ 2 ppm, consistent with a stronger tendency to adopt an α-helical conformation and indicating a more populated or stable helical region within the fragment. Together, these results indicate that BRCA1^467–696^ is largely disordered but retains a populated helical segment between residues 647 and 654, along with several transient secondary structure elements. Future investigations will be necessary to confirm the presence of these structural features and to determine if they form sites important for mediating BRCA1-protein or BRCA1-nucleic acid interactions or have other functions.

## Data Availability

NMRPipe processing scripts available upon reasonable request, expression plasmids deposited with Addgene, resonance assignments deposited in the BMRB (entry 53585).

## Abbreviations

BARD1: BRCA1-associated RING domain protein 1
BRCA1: Breast cancer type 1 susceptibility protein
BRCT: BRCA1 C-terminal
DSS: Sodium trimethylsilylpropanesulfonate
HR: Homologous recombination
HSQC: Heteronuclear single quantum coherence
IDR: Intrinsically disordered region
IPTG: Isopropyl β-D-1-thiogalactopyranoside
LB: Lysogeny broth
NUS: Non-uniform sampling
PMSF: Phenylmethylsulfonyl fluoride
SSP: Secondary structure propensity
TCEP: Tris(2-carboxyethyl)phosphine
